# Identification and Expression Profiles of Sex Pheromone Biosynthesis and Transport Related Genes in *Spodoptera litura*

**DOI:** 10.1371/journal.pone.0140019

**Published:** 2015-10-07

**Authors:** Ya-Nan Zhang, Xiu-Yun Zhu, Li-Ping Fang, Peng He, Zhi-Qiang Wang, Geng Chen, Liang Sun, Zhan-Feng Ye, Dao-Gui Deng, Jin-Bu Li

**Affiliations:** 1 College of Life Sciences, Huaibei Normal University, Huaibei, China; 2 State Key Laboratory Breeding Base of Green Pesticide and Agricultural Bioengineering, Key Laboratory of Green Pesticide and Agricultural Bioengineering, Ministry of Education, Guizhou University, Guiyang, China; 3 Tea Research Institute, Chinese Academy of Agricultural Sciences, Hangzhou, China; 4 Education Ministry, Key Laboratory of Integrated Management of Crop Diseases and Pests, College of Plant Protection, Nanjing Agricultural University, Nanjing, China; United States Department of Agriculture (USDA), UNITED STATES

## Abstract

Although the general pathway of sex pheromone synthesis in moth species has been established, the molecular mechanisms remain poorly understood. The common cutworm *Spodoptera litura* is an important agricultural pest worldwide and causes huge economic losses annually. The female sex pheromone of *S*. *litura* comprises Z9,E11-14:OAc, Z9,E12-14:OAc, Z9-14:OAc, and E11-14:OAc. By sequencing and analyzing the transcriptomic data of the sex pheromone glands, we identified 94 candidate genes related to pheromone biosynthesis (55 genes) or chemoreception (39 genes). Gene expression patterns and phylogenetic analysis revealed that two desaturase genes (*SlitDes5* and *SlitDes11*) and one fatty acyl reductase gene (*SlitFAR3*) showed pheromone gland (PG) biased or specific expression, and clustered with genes known to be involved in pheromone synthesis in other moth species. Furthermore, 4 chemoreception related genes (*SlitOBP6*, *SlitOBP11*, *SlitCSP3*, and *SlitCSP14*) also showed higher expression in the PG, and could be additional candidate genes involved in sex pheromone transport. This study provides the first solid background information that should facilitate further elucidation of sex pheromone biosynthesis and transport, and indicates potential targets to disrupt sexual communication in *S*. *litura* for a novel pest management strategy.

## Introduction

Species-specific sex pheromone-elicited behaviors play a key role in sexual communication and reproduction in most moth species, which therefore serve as a good model to study reproductive isolation in animals, from insects to mammals [1–3]. Moth sex pheromones are biosynthesized and released by specialized sex pheromone glands (PGs) that are located along the inter-segmental membrane between the 8^th^ and 9^th^ abdominal segments of females [4, 5]. In most moths, sex pheromone components are composed of C10 –C18 unsaturated acyclic aliphatic compounds with a functional group such as formyl, hydroxyl, or acyloxyl [6, 7].

Sex pheromone biosynthesis in moths begins with a palmitic or stearic acid moiety that is synthesized *de novo* in the PG through modifications of the fatty acid biosynthetic pathway [8]. Through a series of enzymatic reactions such as desaturation, chain-shortening reaction, reduction, acetylation, and oxidation, the palmitic or stearic acids are then converted to the final pheromone components in a step-wise manner [5, 9, 10]. Therefore, different enzymes are likely to be involved in the different reactions, and to date, the genes encoding 4 different classes of enzymes that are essential for this pathway have been functionally identified—desaturases (Des), fatty acid reductases (FARs), fatty acid transport proteins (FATPs), and acyl-CoaA-binding proteins (ACBPs). Among these, Des proteins are the most intensively studied class of enzymes involved in moth sex pheromone biosynthesis, which can introduce double bonds into pheromone precursors. Previous studies have demonstrated the broad functional diversity of these enzymes such as Δ5 [11, 12], Δ6 [13], Δ9 [9, 14], Δ11 [15, 16], Δ10–12 [10], and Δ14 desaturase [17]. FARs are responsible for reducing fatty acids to alcohols, and have also been functionally identified in a few moth species, including *Bombyx mori* [18], *Ostrinia nubilalis* [19] and 4 *Heliothine* species [20]. FATPs and ACBPs have been functionally identified to play roles in the production of the *B*. *mori* sex pheromone bombykol based on *in vivo* RNA interference methods [21, 22]. In addition, some other important enzymes that have not been functionally confirmed are postulated to be involved in the sex pheromone biosynthesis pathway. For example, biochemical studies have suggested that acetyltransferase (ACT) and alcohol oxidase (AO) play a role by converting alcohol into acetate ester [23] and by oxidizing alcohol into the corresponding aldehyde component [13, 24], respectively.

The common cutworm *Spodoptera litura* (Lepidoptera: Noctuidae) is an important agricultural pest worldwide and causes huge economic losses annually. The female sex pheromones of *S*. *litura* have been identified as a blend of Z9-E11-14:OAc, Z9-E12-14:OAc, Z9-14:OAc, and E11-14:OAc with a ratio of 100:27:20:27 in China [25]. To date, only 9 chemoreception genes have been identified and functionally characterized, including 5 odorant-binding proteins (OBPs) [26, 27] and 4 pheromone receptors (PRs) [28]. However, the genes involved in the pheromone biosynthesis of *S*. *litura* have not been explored. In present study, we constructed a genetic database of the genes expressed in the female PGs of *S*. *litura* using the Illumina HiSeq^(TM)^ 2500 sequencing platform. In total, we identified 94 genes that are possibly related to pheromone biosynthesis (55 genes) or chemoreception (39 genes). Furthermore, tissue expression evaluation and phylogenetic analyses were performed to postulate the functions of the identified genes. The results indicated that some of these genes might play crucial roles in the biosynthesis and transport of *S*. *litura* sex pheromones and could be as candidates for further functional studies.

## Results

### Transcriptome Sequencing and Sequence Assembly

**Fig 1:**
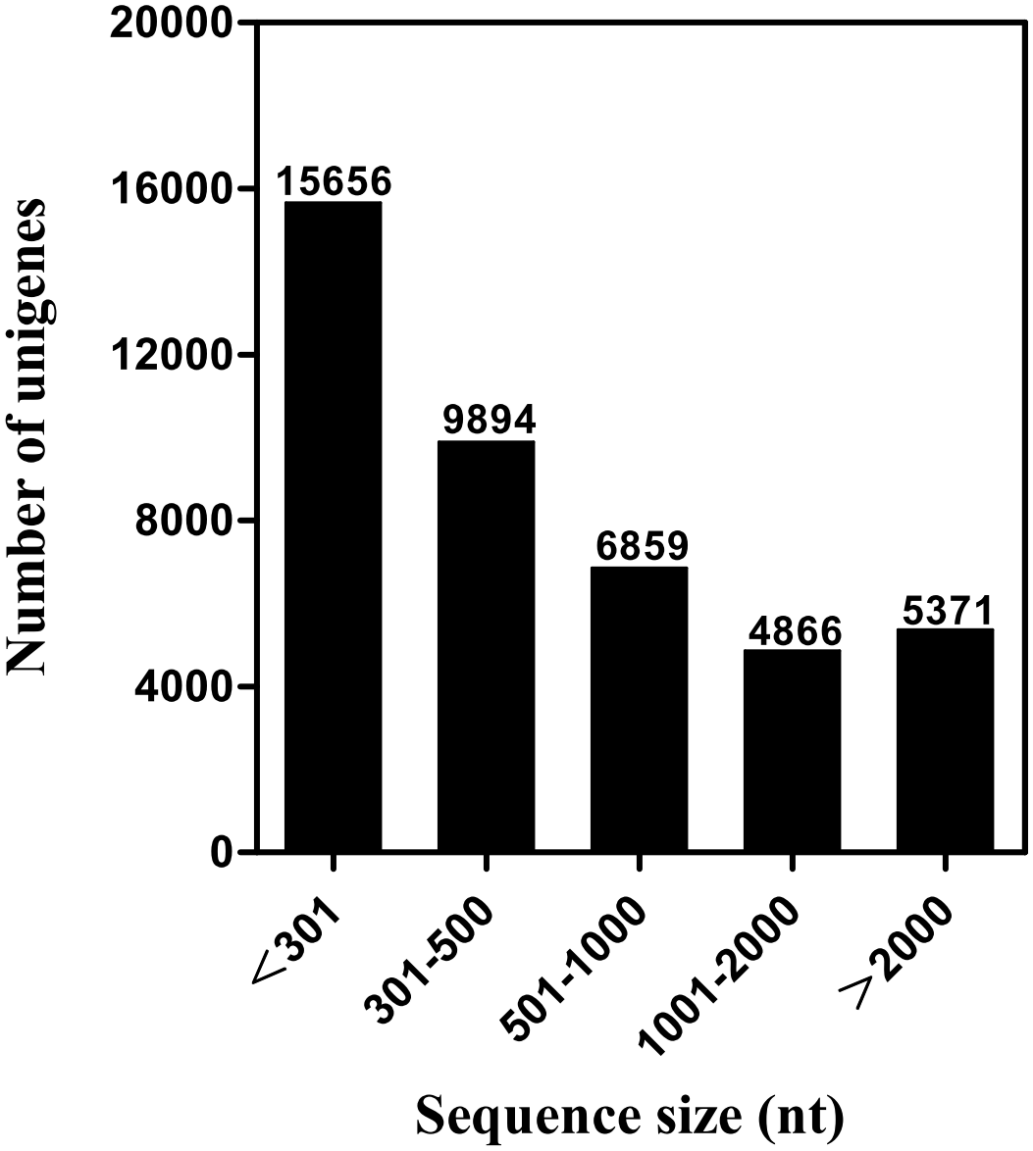
Distribution of unigene size in the *S*. *litura* transcriptome assembly.

We carried out a next-generation sequencing analysis using a cDNA library constructed from the female PGs of *S*. *litura* using the Illumina HiSeq^(TM)^ 2500 platform. The transcriptome sequencing provided approximately 63 million reads (6.3 Gb). After clustering and redundancy filtering, we finally acquired 42,646 unigenes with an N50 length of 2,093 bp (Table 1). We called these 42,646 ones unigenses according to some recently published papers [29, 30], although each of them may not necessarily represents a unique gene. Of the 42,646 unigenes, those with a sequence length of more than 500 bp accounted for 40.08% of the total transcriptome assembly (Fig 1).

**Table 1:** Summary of *S*. *litura* transcriptome assembly.

Statistics Project	Number
Total clean reads	63,209,172
GC percentage	44.69%
Q20 percentage	97.38%
Total unigene nucleotides	39,831,030
Total unigene	42,646
N50 of unigenes (nt)	2,093
Min length of unigenes (nt)	201
Median length of unigenes (nt)	385
Max length of unigenes (nt)	41,274
Unigenes with homolog in NR	14,370

### Gene Ontology (GO) Annotation

**Fig 2:**
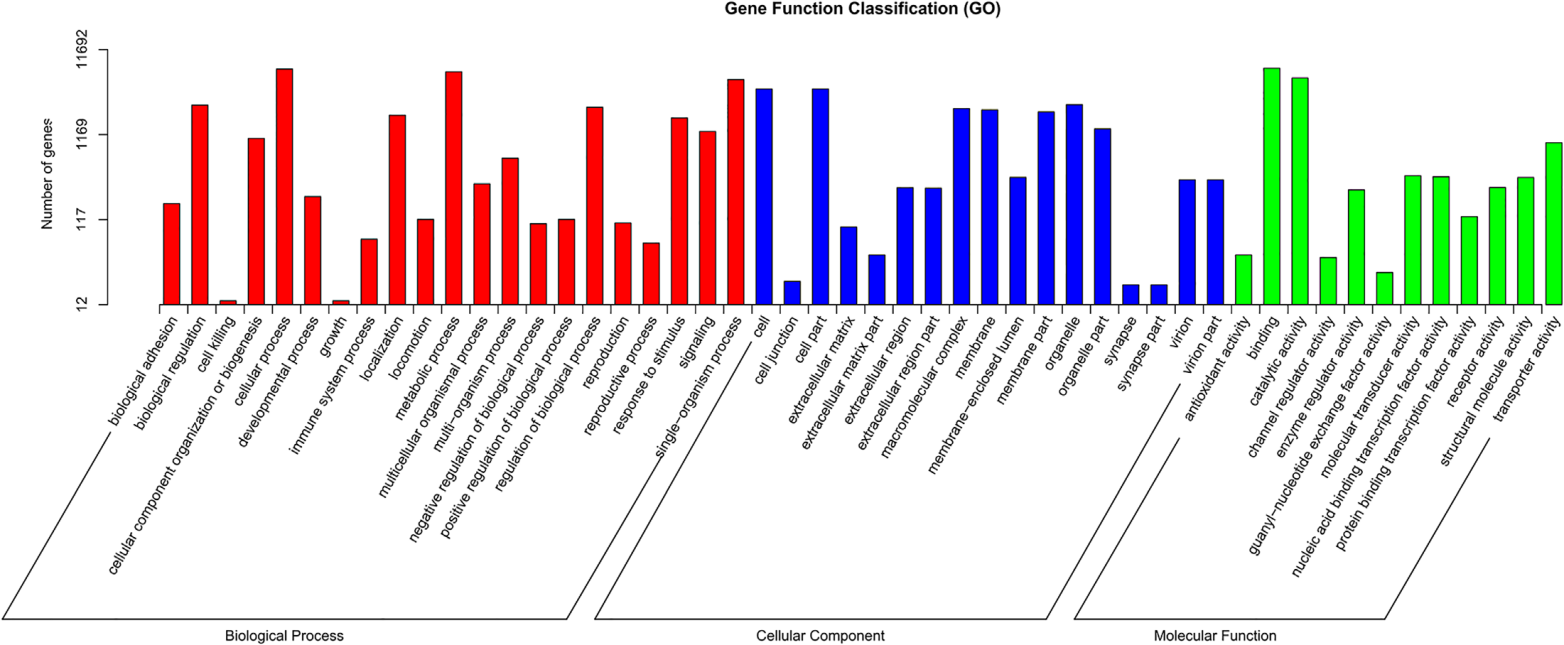
Gene ontology (GO) classification of the *S*. *litura* transcripts with Blast2GO program.

**Fig 3:**
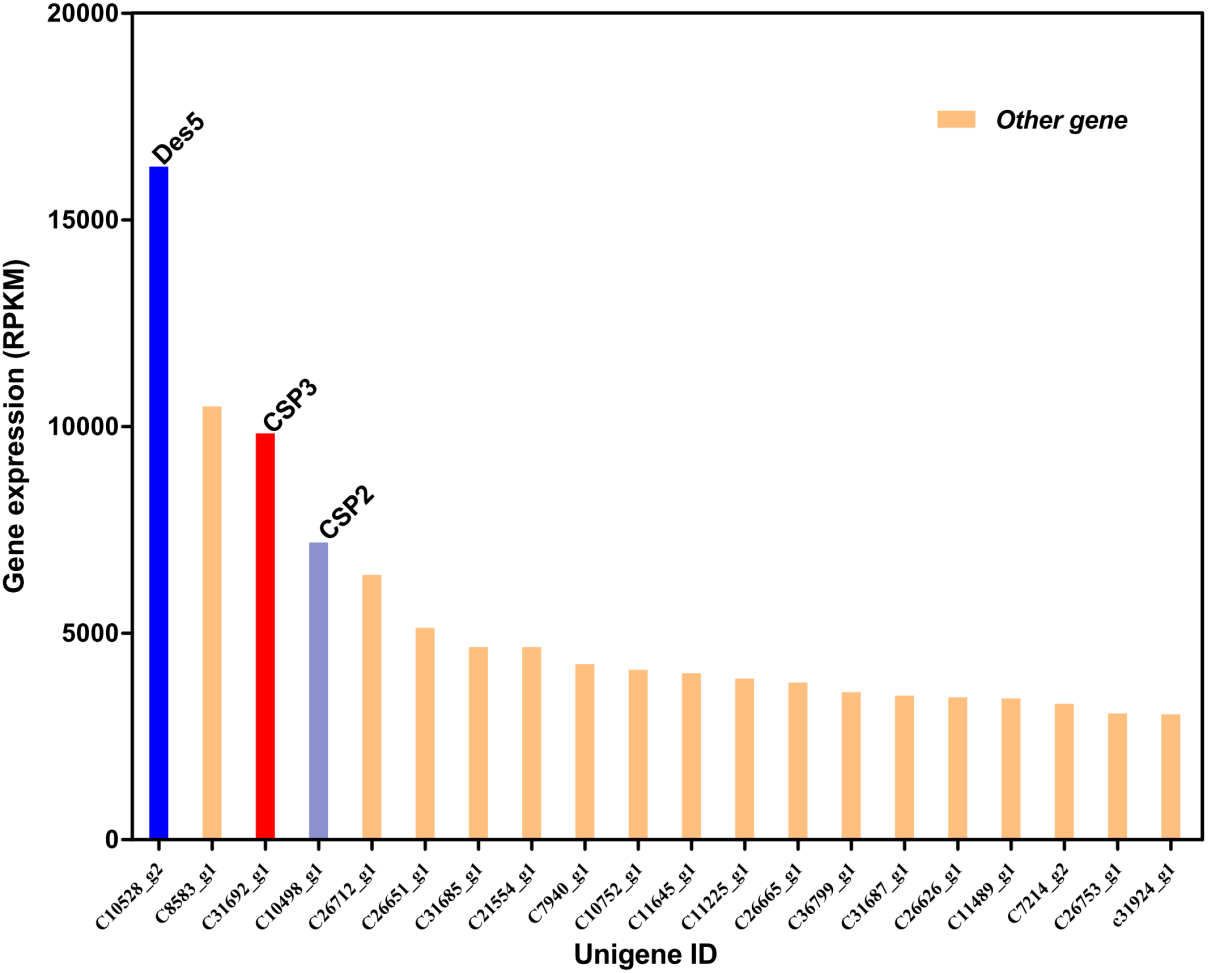
Top 20 most abundant transcripts in the *S*. *litura* transcriptome dataset. The genes expression abundance is indicated as the Reads Per Kilobase per Million mapped reads (RPKM) values. The transcript annotation by homologous comparisons with Blastx is indicated in Tables 3 and 4 and S1 Table.

GO annotation was used to classify the transcripts into functional groups according to GO categories. Of the 42,646 unigenes, 11,692 (27.41%) could be annotated based on sequence homology. In the molecular function category, the genes expressed in the PG were mostly enriched to binding, catalytic activity and transporter activity. Cellular and metabolic processes were the most highly represented in the biological process categories, and cell, cell part, and organelle were most abundantly represented in the cellular component category (Fig 2). In addition, the Des gene *SlitDes5* was the most abundant of all unigenes, and two CSP genes (*SlitCSP3* and *SlitCSP2*) also showed very high abundance (Fig 3 and S1 Table).

### Identification of Putative Genes Related to Sex Pheromone Biosynthesis and Transport

By homology analysis using the reported genes of other moth species in NCBI as queries [31–36], we identified a total of 94 transcripts that belong to gene families that are putatively involved in sex pheromone biosynthesis and transport, including 12 *Des*, 13 *FAR*, 23 *ACT*, 2 *ACBP*, 4 *FATP*, 1 *ACC*, 25 *OBP*, and 14 *CSP* genes. In comparison to sequences of other insects, we found that the number of *Des* genes *in S*. *litura* (12) is close to *H*. *assulta* (8) [35] and *H*. *virescens* (9) [31], but is lower than that of *B*. *mori* (24) (GenBank data) and *Ephestia cautella* (21) [36]. Furthermore, the number of *FAR* genes in *S*. *litura* (13) is the same as that in *H*. *assulta* (13) [35] and *Agrotis ipsilon* (13) [32], but is less than that of *H*. *armigera* (18) [35] and *E*. *cautella* (28) [36]. The numbers of *OBP* (25) and *CSP* genes (14) in *S*. *litura* are similar to those in *H*. *armigera* (26 and 19) [35] and *H*. *assulta* (23 and 16) [35], but are more than those in other moth species [31, 32, 34, 36] (Tables 2–4).

**Table 2:** The number of sex pheromone biosynthesis and transport related genes in different moths.

Species	Sex pheromone biosynthesis						Chemoreception	
	Des	FAR	ACT	ACC	ACBP	FATP	OBP	CSP
*Spodoptera litura*	12 (9)	13 (11)	23 (16)	1 (0)	2 (1)	4 (1)	25 (2)	14 (2)
*Heliothis virescens*	9	5	3	1	—	—	9	16
*Agrotis ipsilon*	5 (2)	13 (3)	5 (3)	2 (2)	—	—	7 (1)	8 (1)
*Chilo suppressalis*	6 (2)	10 (1)	—	1 (0)	3 (0)	4 (0)	9 (0)	10 (2)
*Helicoverpa armigera*	7 (2)	18 (12)	—	2 (1)	—	—	26 (1)	19 (5)
*Helicoverpa assulta*	8 (3)	13 (11)	—	2 (1)	—	—	23 (3)	16 (2)
*Ephestia cautella*	21	18	18	6	—	5	17	7

Note: Digits in parentheses indicate the number of genes with PG-specific or biased expression. “—” indicates the gene has not been reported. The expression profiles of genes in PGs and other tissues of *H*. *virescens* and *E*. *cautella* had not been reported. All of the data are based on PGs transcriptome analyses, and the references are [31, 32, 34, 35, 36].

**Table 3:** The Blastx match of *S*. *litura* sex pheromone biosynthesis genes.

Gene Name	Acc. No.	ORF (aa)	Complete ORF	Best Blastx Match				
				Name	Acc. No.	Species	E value	Identity (%)
***Desaturase (Des)***								
Des1	KT261685	38	N	acyl-CoA delta 11 desaturaseacyl	AGR49312.1	*Agrotis ipsilon*	3.00E-11	54
Des2	KT261686	376	Y	desaturase	AAQ74260.1	*Spodoptera littoralis*	0.00E+00	99
Des3	KT261687	444	Y	desaturase	AID66662.1	*Agrotis segetum*	0.00E+00	80
Des4	KT261688	335	Y	desaturase	AID66661.1	*Agrotis segetum*	0.00E+00	90
Des5	AGH12217.1	338	Y	delta 11 desaturase	AGH12217.1	*Spodoptera litura*	0.00E+00	100
Des6	KT261689	50	N	delta 11 desaturase	AGH12217.1	*Spodoptera litura*	2.00E-28	68
Des7	KT261690	84	N	delta–9 desaturase 14–26	AFO38465.1	*Spodoptera exigua*	1.00E-79	100
Des8	KT261691	321	Y	desaturase	AID66658.1	*Agrotis segetum*	0.00E+00	93
Des9	AGH12218.1	353	Y	delta 9 desaturase	AGH12218.1	*Spodoptera litura*	0.00E+00	100
Des10	KT261692	451	Y	desaturase	AID66663.1	*Agrotis segetum*	0.00E+00	90
Des11	KT261693	353	Y	delta–9 desaturase	AAQ74258.1	*Spodoptera littoralis*	0.00E+00	99
Des12	KT261694	296	N	acyl-CoA Delta(11)	NP_001274329.1	*Bombyx mori*	2.00E-158	73
***Fatty-Acyl Reductase (FAR)***								
FAR1	KT261695	535	Y	fatty acyl reductase	AID66649.1	*Agrotis segetum*	9.10E-80	84
FAR2	KT261696	234	N	fatty-acyl CoA reductase 6	XP_004923270.1	*Agrotis ipsilon*	2.00E-133	87
FAR3	KT261697	454	Y	Far1 protein	CDG50833.1	*Spodoptera littoralis*	0.00E+00	99
FAR4	KT261698	250	N	fatty acyl reductase	AID66654.1	*Agrotis segetum*	3.00E-164	90
FAR5	KT261699	522	N	fatty acyl-CoA reductase CG5065-like	XP_004925987.1	*Bombyx mori*	0.00E+00	49
FAR6	KT261700	520	Y	fatty-acyl CoA reductase 6	AGR49316.1	*Agrotis ipsilon*	0.00E+00	67
FAR7	KT261701	520	Y	fatty acyl-CoA reductase CG5065-like	XP_004929961.1	*Bombyx mori*	0.00E+00	78
FAR8	KT261702	526	Y	fatty acyl reductase	AID66652.1	*Agrotis segetum*	0.00E+00	89
FAR9	KT261703	231	N	fatty acyl reductase	AID66654.1	*Agrotis segetum*	5.00E-158	88
FAR10	KT261704	624	Y	fatty acyl reductase	AID66650.1	*Agrotis segetum*	0.00E+00	85
FAR11	KT261705	512	Y	fatty acyl reductase	AID66647.1	*Agrotis segetum*	0.00E+00	55
FAR12	KT261706	510	Y	fatty acyl-CoA reductase	AGR49318.1	*Agrotis ipsilon*	0.00E+00	90
FAR13	KT261707	246	N	fatty acyl reductase	AID66647.1	*Agrotis segetum*	0.00E+00	80
***Acetyltransferase (ACT)***								
ACT1	KT261708	396	Y	fatty alcohol acetyltransferase	AIN34689.1	*Agrotis segetum*	0.00E+00	94
ACT2	KT261709	383	Y	fatty alcohol acetyltransferase	AIN34698.1	*Agrotis segetum*	0.00E+00	96
ACT3	KT261710	719	Y	fatty alcohol acetyltransferase	AIN34709.1	*Agrotis segetum*	0.00E+00	65
ACT4	KT261711	271	Y	acyltransferase AGPAT2	AGG54993.1	*Heliothis virescens*	9.00E-178	90
ACT5	KT261712	397	Y	fatty alcohol acetyltransferase	AIN34682.1	*Agrotis segetum*	0.00E+00	73
ACT6	KT261713	714	Y	fatty alcohol acetyltransferase	AIN34685.1	*Agrotis segetum*	0.00E+00	99
ACT7	KT261714	276	N	fatty alcohol acetyltransferase	AIN34682.1	*Agrotis segetum*	1.00E-165	90
ACT8	KT261715	180	Y	acetyltransferase	AGQ45625.1	*Agrotis ipsilo*	1.00E-124	97
ACT9	KT261716	471	Y	fatty alcohol acetyltransferase	AIN34706.1	*Agrotis segetum*	9.00E-177	96
ACT10	KT261717	479	Y	fatty alcohol acetyltransferase	AIN34712.1	*Agrotis segetum*	0.00E+00	94
ACT11	KT261718	431	Y	fatty alcohol acetyltransferase	AIN34699.1	*Agrotis segetum*	0.00E+00	95
ACT12	KT261719	477	Y	fatty alcohol acetyltransferase	AIN34710.1	*Agrotis segetum*	0.00E+00	84
ACT13	KT261720	373	N	fatty alcohol acetyltransferase	AIN34702.1	*Agrotis segetum*	4.00E-25	71
ACT14	KT261721	652	Y	fatty alcohol acetyltransferase	AIN34708.1	*Agrotis segetum*	0.00E+00	89
ACT15	KT261722	95	N	fatty alcohol acetyltransferase	AIN34682.1	*Agrotis segetum*	3.00E-52	85
ACT16	KT261723	282	Y	acyltransferase AGPAT5	AGG55013.1	*Heliothis subflexa*	4.00E-166	89
ACT17	KT261724	359	Y	fatty alcohol acetyltransferase	AIN34693.1	*Agrotis segetum*	0.00E+00	89
ACT18	KT261725	355	Y	fatty alcohol acetyltransferase	AIN34705.1	*Agrotis segetum*	0.00E+00	90
ACT19	KT261726	390	Y	fatty alcohol acetyltransferase	AIN34704.1	*Agrotis segetum*	0.00E+00	88
ACT20	KT261727	330	Y	fatty alcohol acetyltransferase	AIN34713.1	*Agrotis segetum*	0.00E+00	83
ACT21	KT261728	400	Y	fatty alcohol acetyltransferase	AIN34683.1	*Agrotis segetum*	0.00E+00	92
ACT22	KT261729	480	Y	fatty alcohol acetyltransferase	AIN34694.1	*Agrotis segetum*	0.00E+00	83
ACT23	KT261730	504	Y	fatty alcohol acetyltransferase	EFN73032.1	*Agrotis segetum*	4.00E-128	47
***Acetyl-CoA Carboxylase (ACC)***								
ACC	KT261731	2385	Y	acetyl-CoA carboxylase-like	AID66639.1	*Agrotis segetum*	0.00E+00	94
***Fatty Acid Transport Protein (FATP)***								
FATP1	KT261734	700	Y	fatty acid transport protein 1	AII21952.1	*Sesamia inferens*	0.00E+00	90
FATP2	KT261735	651	Y	fatty acid transport protein 2	AII21953.1	*Sesamia inferens*	0.00E+00	89
FATP3	KT261736	661	Y	fatty acid transport protein 3	AII21954.1	*Sesamia inferens*	0.00E+00	86
FATP4	KT261737	643	Y	fatty acid transport protein 4	AII21955.1	*Sesamia inferens*	0.00E+00	93
***Acyl-CoA Binding Protein (ACBP)***								
ACBP1	KT261732	255	Y	acyl-CoA binding protein 3	AII21948.1	*Sesamia inferens*	8.00E-141	83
ACBP2	KT261733	90	Y	acyl-CoA binding protein 1	AII21946.1	*Sesamia inferens*	1.00E-45	92

**Table 4:** The Blastx match of *S*. *litura* chemoreception genes.

Gene Name	Acc. No.	ORF (aa)	Signal Peptide	Complete ORF	Best Blastx Match				
					Name	Acc. No.	Species	E value	Identity (%)
***Odorant Binding Protein (OBP)***									
OBP1	KT261647	147	1–20	Y	odorant binding protein 6	AFM77984.1	*Spodoptera exigua*	9.10E-80	78
OBP2	KT261648	156	1–18	Y	odorant-binding protein 2	XP_004923270.1	*Chilo suppressalis*	2.00E-16	31
OBP3	KT261649	104	1–17	N	odorant binding protein 11	AGH70107.1	*Spodoptera exigua*	2.00E-13	100
OBP4	KT261650	167	1–16	Y	odorant binding protein	AII00985.1	*Dendrolimus houi*	2.00E-07	32
OBP5	KT261651	145	1–24	Y	SexiOBP13	AGP03459.1	*Spodoptera exigua*	4.00E-79	84
OBP6	KT261652	158	1–26	Y	SexiOBP8	AGP03454.1	*Spodoptera exigua*	2.00E-42	58
OBP7	KT261653	213	1–18	Y	odorant-binding protein 19	AGC92793.1	*Helicoverpa assulta*	3.00E-56	54
OBP8	KT261654	184	1–20	Y	odorant binding protein	AII00978.1	*Dendrolimus houi*	7.00E-109	92
OBP9	KT261655	239	1–19	Y	odorant binding protein fmxg18C17	NP_001157372.1	*Bombyx mori*	1.00E-70	50
OBP10	KT261656	115	N	N	odorant-binding protein 1	AFG72998.1	*Cnaphalocrocis medinalis*	8.00E-71	83
OBP11	KT261657	149	1–21	Y	SexiOBP8	AGP03454.1	*Spodoptera exigua*	4.00E-10	35
OBP12	KT261658	74	N	N	SexiOBP14	AGP03460.1	*Spodoptera exigua*	3.00E-46	90
OBP13	KT261659	129	1–17	Y	odorant binding protein	ADY17884.1	*Spodoptera exigua*	1.00E-56	81
OBP14	KT261660	193	1–17	Y	odorant binding protein 1	AGR39564.1	*Agrotis ipsilon*	1.00E-72	58
OBP15	KT261661	147	1–17	Y	odorant binding protein 6	AGR39569.1	*Agrotis ipsilon*	8.00E-60	76
OBP16	KT261662	137	1–19	Y	antennal binding protein X	CAA05508.1	*Heliothis virescens*	1.00E-62	92
OBP17	KT261663	147	1–21	Y	antennal binding protein	ADY17881.1	*Spodoptera exigua*	9.00E-90	91
OBP18	KT261664	139	1–18	Y	OBP8	AEB54589.1	*Helicoverpa armigera*	1.00E-81	84
OBP19	KT261665	52	N	N	odorant binding protein 11	AGH70107.1	*Spodoptera exigua*	1.00E-32	92
OBP20	KT261666	147	1–21	Y	odorant-binding protein	AAR28762.1	*Spodoptera exigua*	1.00E-78	93
OBP21	KT261667	240	1–20	Y	odorant binding protein fmxg18C17	NP_001157372.1	*Bombyx mori*	2.00E-37	43
OBP22	KT261668	133	1–16	Y	odorant binding protein 9	AGH70105.1	*Spodoptera exigua*	1.00E-85	95
OBP23	KT261669	142	1–21	Y	odorant binding protein 7	AGH70103.1	*Spodoptera exigua*	6.00E-93	97
OBP24	KT261670	83	1–24	N	SexiOBP12	AGP03458.1	*Spodoptera exigua*	9.00E-42	88
OBP25	AIS72934.1	148	N	Y	pheromone-binding protein 3	AIS72934.1	*Spodoptera litura*	3.00E-116	100
***Chemosensory Protein (CSP)***									
CSP1	KT261672	122	1–16	Y	chemosensory protein 10	AFR92094.1	*Helicoverpa armigera*	1.00E-63	91
CSP2	KT261673	128	1–18	Y	chemosensory protein CSP2	ABM67689.1	*Spodoptera exigua*	3.00E-71	96
CSP3	KT261674	123	1–18	Y	chemosensory protein 8	AGR39578.1	*Agrotis ipsilon*	1.00E-70	89
CSP4	KT261675	287	1–16	Y	chemosensory protein	AIW65104.1	*Helicoverpa armigera*	9.00E-125	83
CSP5	KT261676	128	1–18	Y	chemosensory protein CSP1	ABM67688.1	*Spodoptera exigua*	7.00E-82	94
CSP6	KT261677	123	1–16	Y	chemosensory protein	AIW65100.1	*Helicoverpa armigera*	2.00E-59	81
CSP7	KT261678	113	1–16	Y	sensory appendage protein-likeprotein	AAK14793.1	*Mamestra brassicae*	2.00E-37	62
CSP8	KT261679	128	1–16	Y	chemosensory protein	AIU68827.1	*Chilo auricilius*	1.00E-83	98
CSP9	KT261680	107	1–18	Y	chemosensory protein 5	AGR39575.1	*Agrotis ipsilon*	4.00E-53	93
CSP10	KT261681	127	1–17	Y	chemosensory protein 6	AGR39576.1	*Agrotis ipsilon*	3.00E-60	87
CSP11	KT261682	78	1–19	N	chemosensory protein 13	BAG71921.1	*Papilio xuthus*	5.00E-32	77
CSP12	KT261683	120	1–16	Y	CSP2	AEX07265.1	*Helicoverpa armigera*	9.00E-75	91
CSP13	KT261684	123	1–18	Y	chemosensory protein 8	AFR92092.1	*Helicoverpa armigera*	2.00E-37	67
CSP14	AAY26143.1	126	1–16	Y	chemosensory protein CSP	AAY26143.1	*Spodoptera litura*	2.00E-75	100

Of the 94 identified genes (Tables 3 and 4), the sequences of 5 genes were identical to those already deposited in GenBank: 3 *SlitDes* genes (GenBank Accession No.: AGH12217.1, AGH12217.1 and AGH12218.1), 1 *SlitOBP* (GenBank Accession No.: AIS72934.1), and 1 *SlitCSP* (GenBank Accession No.: AAY26143.1), whereas the other 89 transcripts found in the current study were new in *S*. *litura*.

### Expression Profile of the Putative Genes Related to Sex Pheromone Biosynthesis and Transport

**Fig 4:**
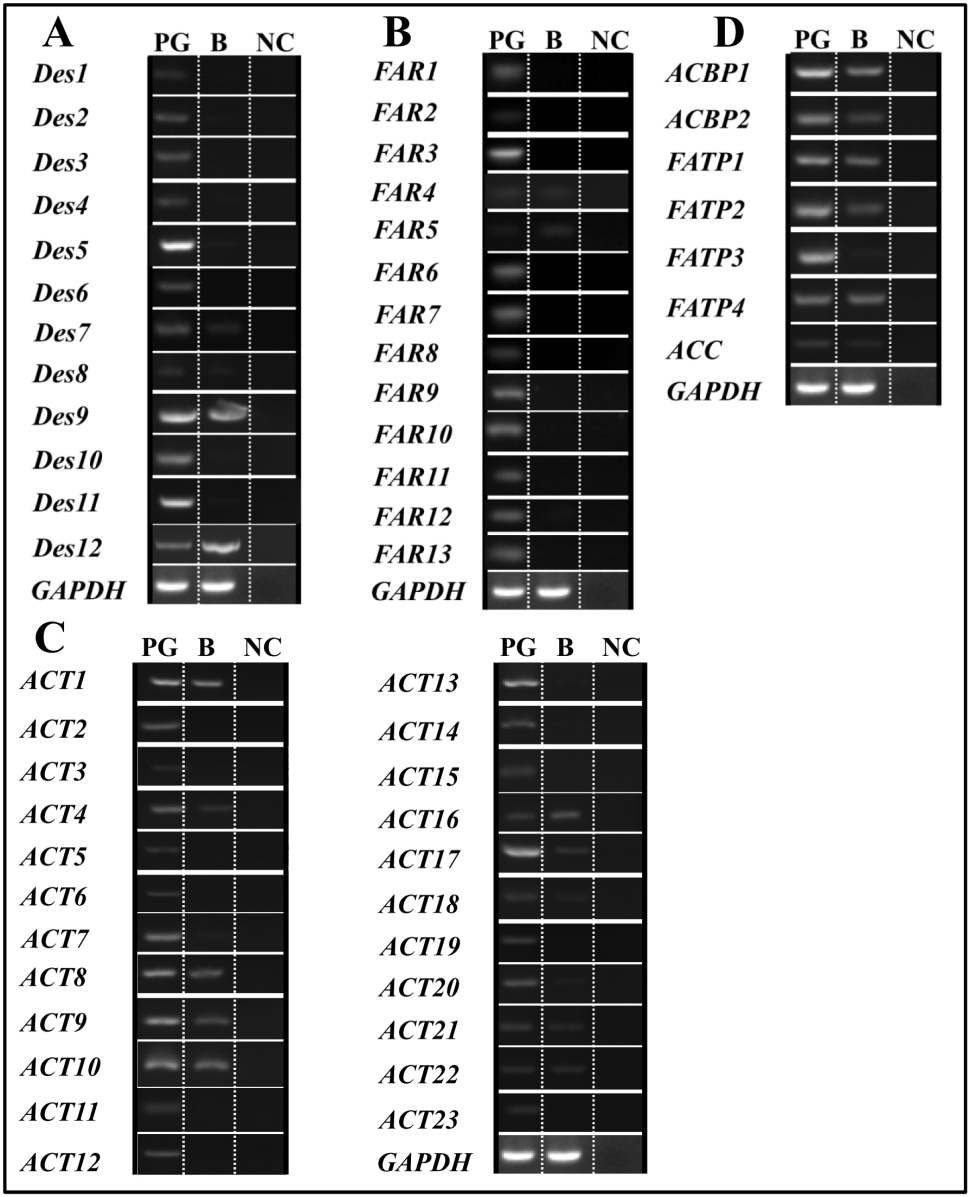
Expression patterns of sex pheromone biosynthesis related genes, using RT-PCR. (A) Expression of Des genes. (B) Expression of FAR genes. (C) Expression of ACT genes. (D) Expression of ACBP, FATP and ACC genes. GAPDH gene was used as a positive control and NC (no cDNA template) as a negative control. PG, female pheromone glands; B, whole insect body without PGs.

**Fig 5:**
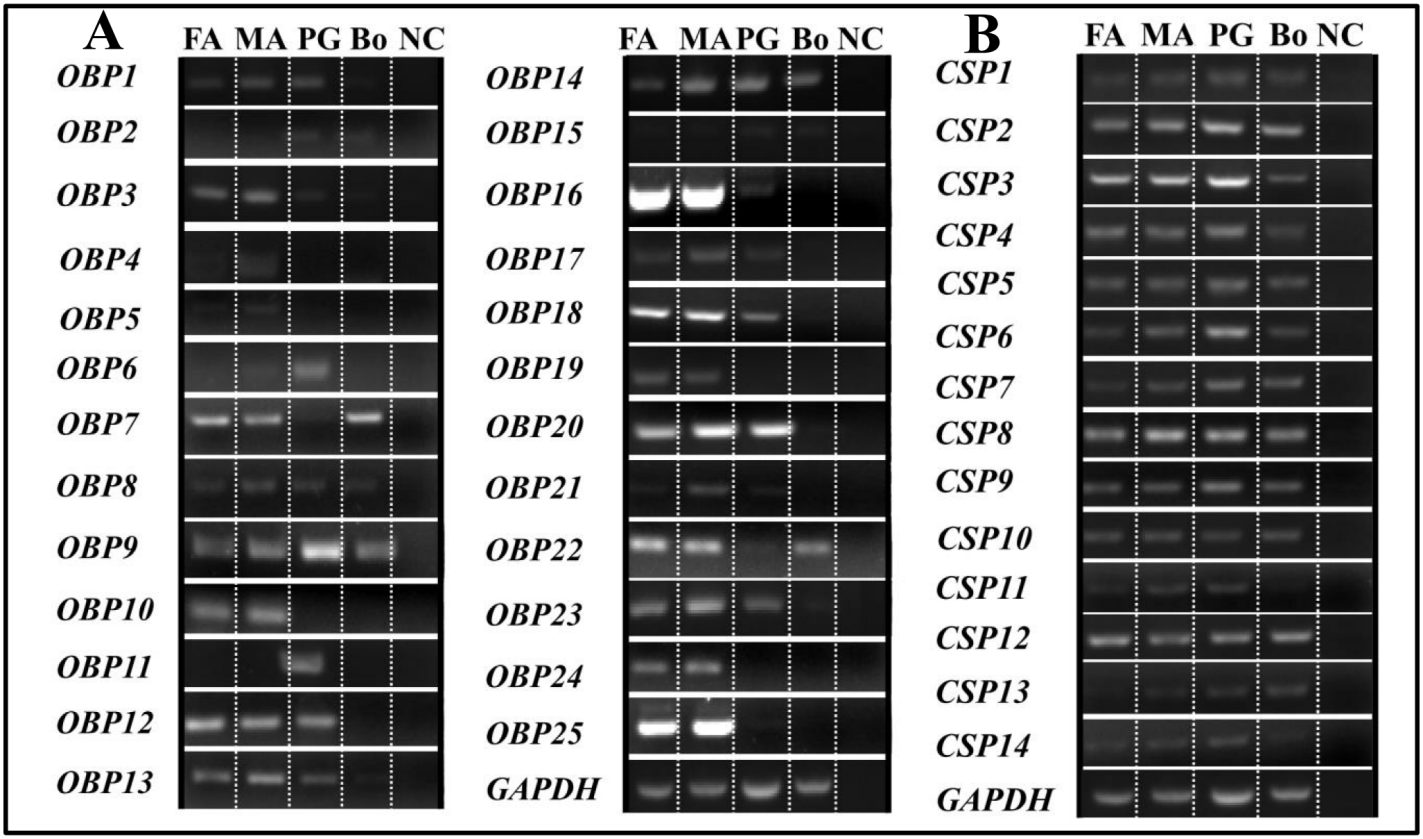
Expression patterns of sex pheromone chemoreception related genes, using RT-PCR. (A) Expression of OBP genes. (B) Expression of CSP genes. GAPDH gene was used as a positive control and NC (no cDNA template) as a negative control. PG, female pheromone glands; B, whole insect body without PGs.

**Fig 6:**
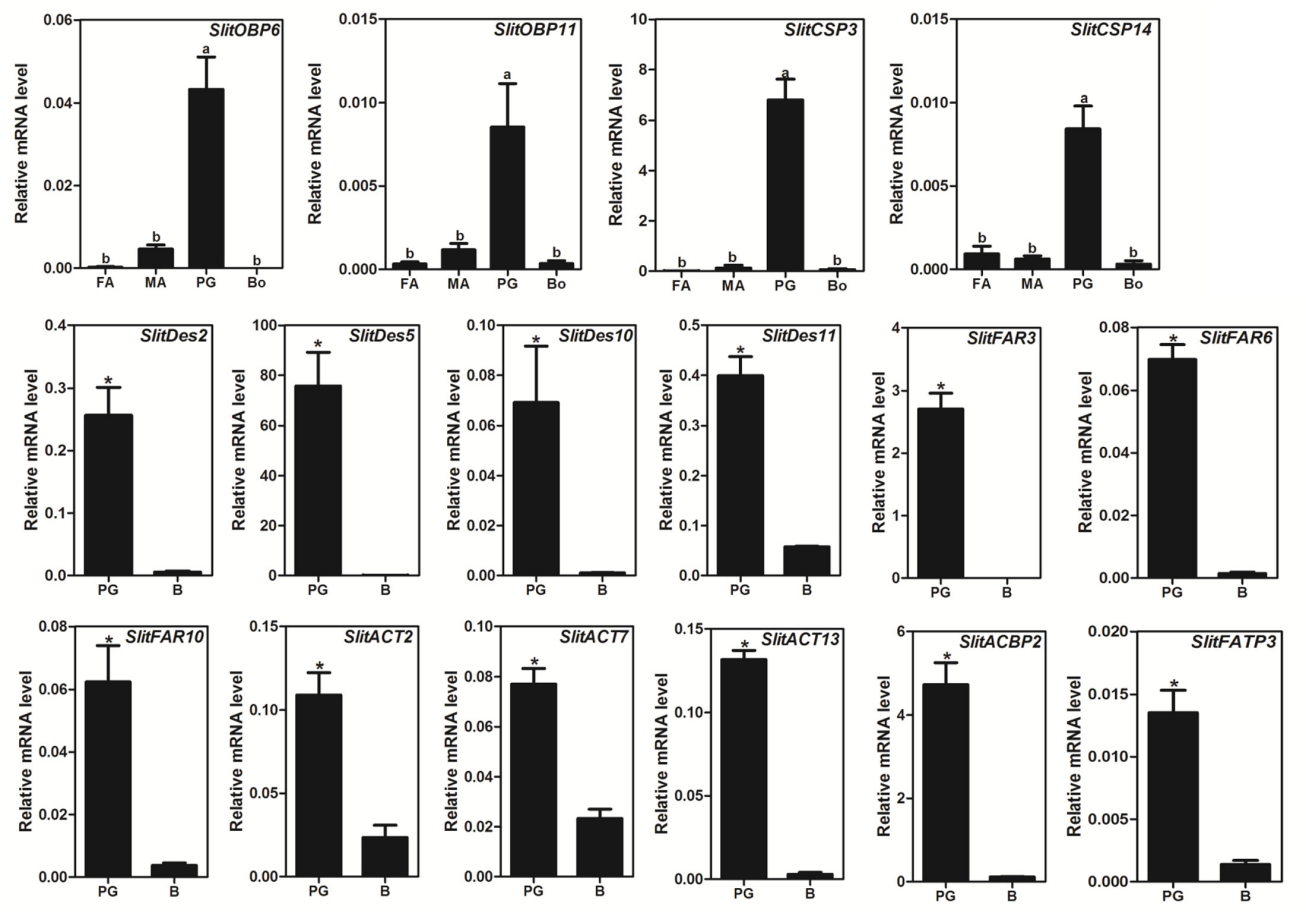
Relative expression levels of 16 pheromone biosynthesis and chemoreception releated genes, using qPCR. FA, female antennae; MA, male antennae; PG, female pheromone glands; Bo, whole insect body without PGs and antennae. The relative expression level is indicated as mean ± SE (N = 3). Different capital letters mean significant difference between tissues (P < 0.05, ANOVA, LSD); the “*” indicates significant difference between male and female (P < 0.05, Student *t*-test).

To investigate the general expression profiles of the candidate genes, reverse transcription-polymerase chain reaction (RT-PCR) analyses were conducted for all 94 genes (Figs 4 and 5), and the expression levels of 16 selected genes were further quantified with qPCR (Fig 6) to validate the RT-PCR results. The overall relative expression profiles of these genes in different tissues obtained were similar with the two methods.

Most of the pheromone biosynthesis-related genes (>70%) displayed PG-biased or specific expression, and 4 chemoreception related genes (*SlitOBP6*, *SlitOBP11*, *SlitCSP3*, and *SlitCSP14*) were also more highly expressed in the PG than in other tissues of *S*. *litura* (Table 2).

#### Desaturase (Des) and Fatty Acyl Reductase (FAR)

The results of RT-PCR and qPCR showed that 9 of the 12 *Des* genes showed PG-biased or specific expression, which is greater than the proportion reported for other moth species (Table 2); the other *Des* genes, *SlitDes7*, *SlitDes9*, and *SlitDes12*, were detected in both the PG and other parts of the body (Table 2, Figs 4A and 6). Similar to *H*. *assulta* [35], 11 of the 13 *SlitFAR* genes (except for *SlitFAR4* and *SlitFAR5*) displayed PG-biased or specific expression (Table 2, Figs 4B and 6).

#### Acetyltransferases (ACT)

Over half of the 23 newly identified *SlitACT* genes were predominantly or specifically expressed in the PG. *SlitACT16* expression was detected to be highly biased in the body, whereas 6 *ACT* genes (*SlitACT1*, *8*, *9*, *10*, *21*, and *22*) displayed similar expression levels in the PG and body (Table 2, Figs 4C and 6).

#### Acyl-CoA Binding Protein (ACBP), Fatty Acid Transport Protein (FATP) and Acetyl-CoA Carboxylase (ACC)

Of the 2 *SlitACBP* and 3 *SlitFATP* genes, only *SlitACBP2* and *SlitFATP3* were highly expressed in the PG, whereas the *SlitACC* gene encoding an ACC with 94% identity to the ACC of *Agrotis segetum* (GenBank Accession No.: AID66639.1) did not show PG-biased or specific expression (Table 2, Figs 4D and 6).

#### Odorant Binding Protein (OBP) and Chemosensory Protein (CSP)

We identified a total of 25 *OBP* genes from the PG of *S*. *litura* in this study, including 1 *PBP* (*OBP25*) and 24 other *OBPs*. Of the 25 *SlitOBPs*, 4 genes (*SlitOBP8*, *SlitOBP9*, *SlitOBP13*, and *SlitOBP14*) displayed a very wide range of tissue distribution in all 4 tissues examined, whereas 8 other genes (*SlitOBP3*, *SlitOBP4*, *SlitOBP5*, *SlitOBP10*, *SlitOBP16*, *SlitOBP19*, *SlitOBP24*, and *SlitOBP25*) were expressed predominately or specifically in the adult antennae. Furthermore, 2 genes (*SlitOBP6 and SlitOBP11*) were expressed at much higher levels in the PG than in other tissues.

Unlike the *SlitOBPs*, most of the *SlitCSPs* were expressed at similar levels among the 4 tissues examined; only *SlitCSP3* and *SlitCSP14* showed significantly higher expression in the PG than in other tissues (Table 2, Figs 5 and 6).

### Phylogenetic Analyses

**Fig 7:**
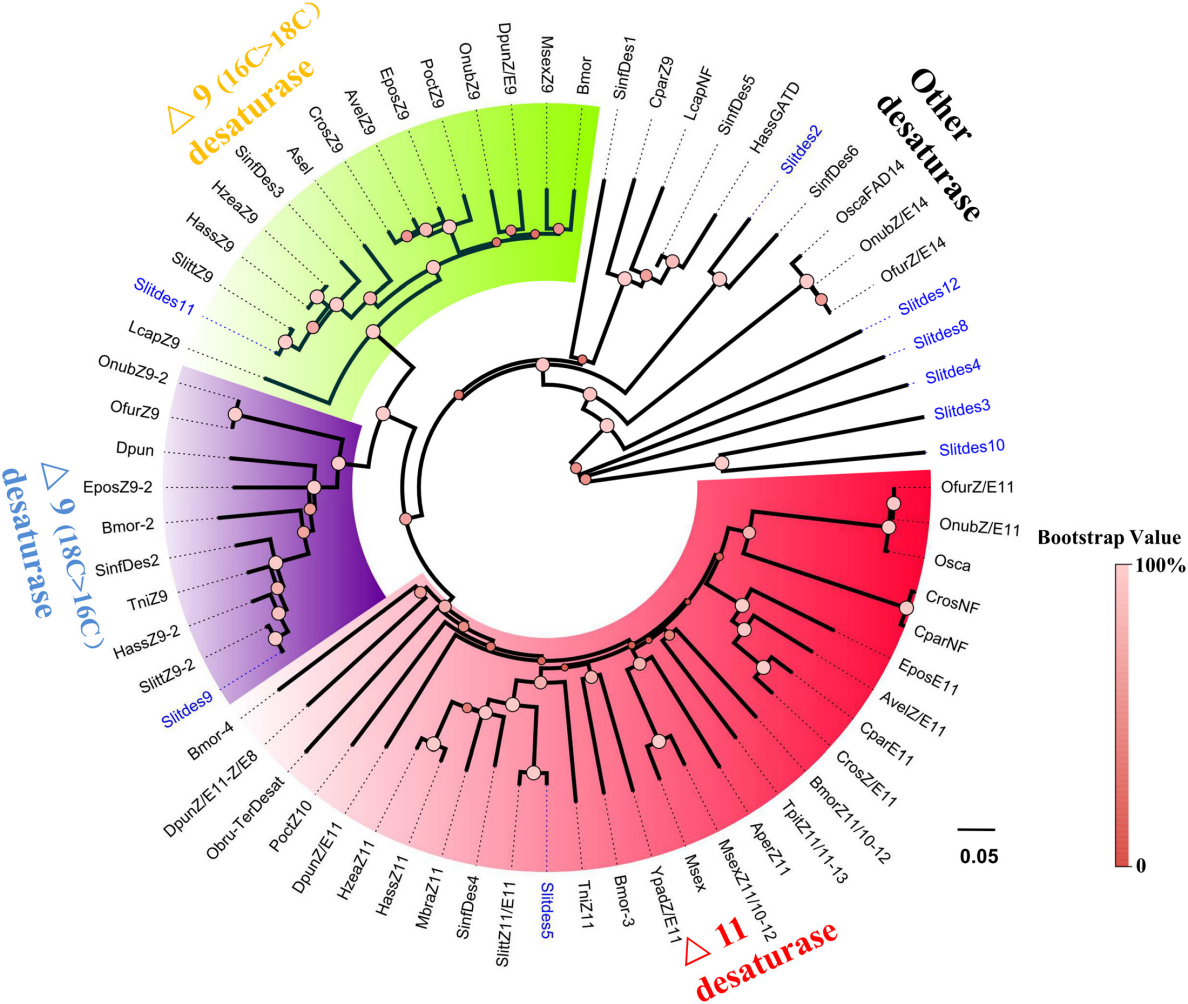
Phylogenetic tree of insect desaturase (Des). The *S*. *litura* translated genes are shown in blue. Accession numbers are given in S2 Table. The tree was constructed with MEGA5.0, using the neighbour-joining method. Values at the nodes are results of bootstrap with 1000 replicates.

**Fig 8:**
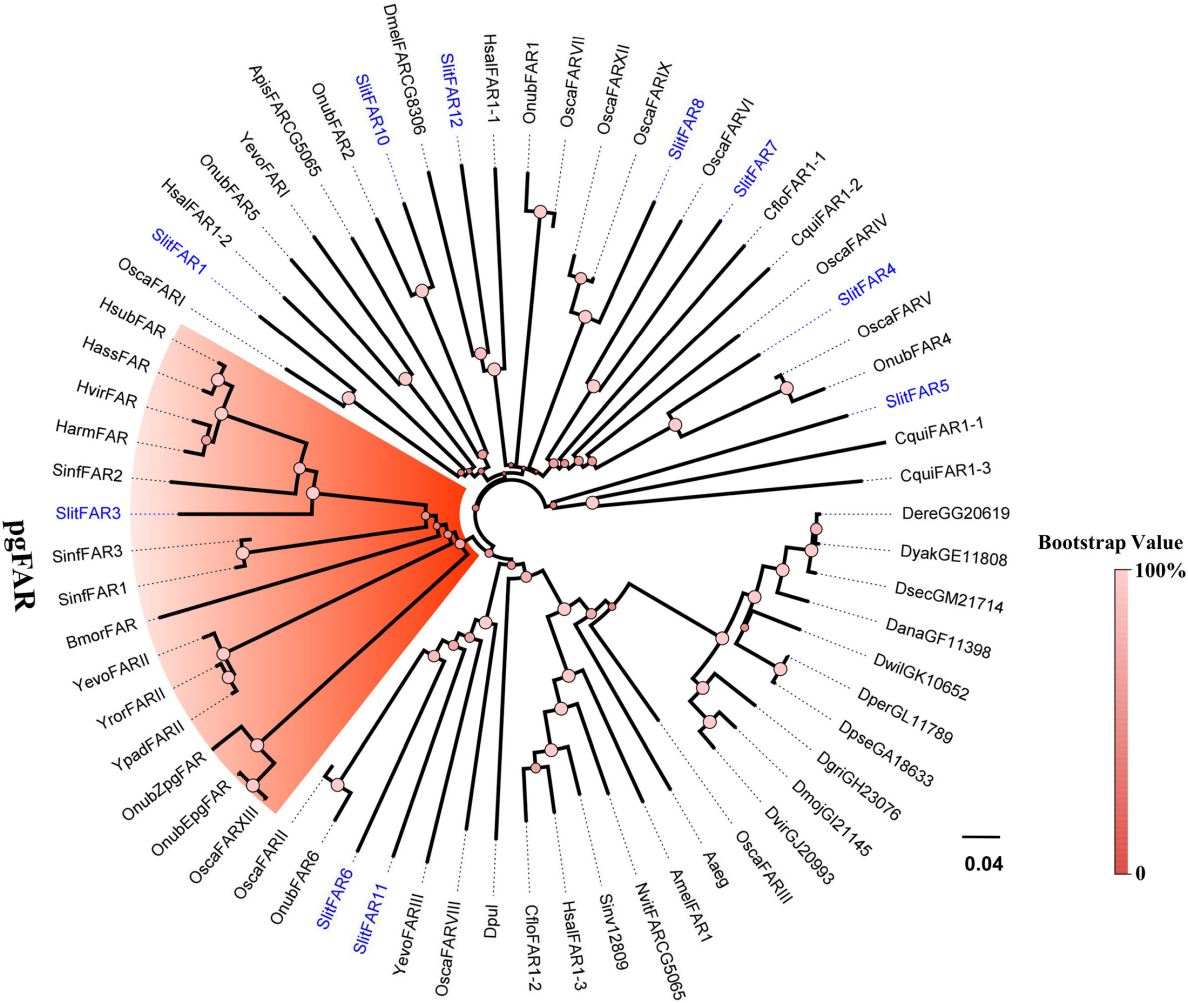
Phylogenetic tree of insect fatty acid redutase (FAR). The *S*. *litura* translated genes are shown in blue. Accession numbers are given in S2 Table. The tree was constructed with MEGA5.0, using the neighbour-joining method. Values at the nodes are results of bootstrap with 1000 replicates.

To assign putative functions to these different genes, phylogenetic analyses were conducted for each group of the enzymes. A phylogenetic tree of Des sequences (Fig 7) showed that 3 SlitfDes genes clearly clustered in 3 different groups of insect desaturases: Δ11-desaturase (SlitDes5), Δ9-desaturase (18C>16C) (SinfDes9), and Δ9-desaturase (16C>18C) (SlitDes11). In the FAR phylogenetic tree, only SlitFAR3 clustered within the lepidopteran pgFAR group that contains previously identified FARs known to be involved in moth sex pheromone biosynthesis [20] (Fig 8).

The OBP phylogenetic tree showed that SlitOBP25 (SlitPBP3) was clustered into the PBP/GOBP clade. The other SlitOBPs and all SlitCSPs were clustered with at least one lepidopteran orthologous gene (S1 and S2 Figs).

## Discussion

In the present study, we sequenced and analyzed the transcriptome of the PG of *S*. *litura*. Among the 42,646 unigenes identified, only 27.41% could be annotated to one or more GO terms, which is similar to other lepidopteran species [37–39], indicating that a large number of *S*. *litura* genes are either non-coding or are homologous with genes that do not have any GO term. Importantly, we identified 89 novel genes that are involved in sex pheromone biosynthesis and transport in *S*. *litura*. Our results not only provide an important foundation for further elucidation of the molecular mechanisms of sex pheromone metabolism but also provide general insight into insect physiology and development of a novel pest control strategy [40].

*S*. *litura* has 4 sex pheromone components: Z9,E11-14:OAc, Z9,E12-14:OAc, Z9-14:OAc, and E11-14:OAc (at a ratio of 100:27:20:27) in China [25]. According to some previous studies related to the biosynthesis pathway of Δ11- or Δ9-containing sex pheromones in several moth species, the defined pathway involves a step of Δ11 or Δ9 desaturation that is catalyzed by Δ11 or Δ9 desaturase, respectively [16, 41–44]. On the other hand, Liu et al. [45] reported that a Δ9 desaturase participates in the production of the Δ11-containing pheromone component by introducing a Δ9-double bond at 14:CoA, followed by carbon chain elongation to Δ11–16:CoA. In sum, these findings indicate that the Δ11 and Δ9 desaturases are likely responsible for introduction of the Δ11 and Δ9-double bonds in all 4 pheromones of *S*. *litura*. In this study, we obtained 9 desaturase genes showing PG-biased or specific expression. Further phylogenetic analysis showed that *SlitDes5* was clearly assigned to the Δ11 desaturase group, and was most closely related to *Z/E11* of *S*. *littoralis* (GenBank Accession No. Q6US81). *SlitDes9* and *SlitDes11* were allocated to the Δ9 (18C>16C) and Δ9 (16C>18C) desaturase groups, respectively, but only *SlitDes11* displayed a PG-biased expression pattern; the other genes were allocated to other desaturase groups. Therefore, *SlitDes5* and *SlitDes11* are very likely involved in the desaturation step from saturated acids (14C) to unsaturated acids, with a double bond introduced at the 11^th^ and 9^th^ positions of the carbon chain, respectively. To date, there is no report on Δ12 desaturase genes in moths; therefore, the type of enzyme that *S*. *litura* uses to introduce a double bond at the 12^th^ position needs to be further studied.

In the process of sex pheromone biosynthesis, once the specific unsaturated fatty acid precursors are produced, they will be converted into corresponding alcohols by FAR, which has been demonstrated in different moth species [18–20, 46]. In this study, we identified 11 *FAR* genes with PG-biased or specific expression, but only *SlitFAR3* was clustered in the moth pgFAR group, suggesting that this gene plays a crucial role in the biosynthesis of precursor alcohols. ACTs are essential in the biosynthesis of acyloxyl components, although no related gene had been functionally characterized to date. In the present study, 15 *ACT* genes were found to be highly or specifically expressed in the PG by RT-PCR and qPCR, indicating that these genes may participate in the progress of ACT to produce corresponding acetate ester. FATPs have been functionally confirmed to bind to and transport fatty acids across the insect hemolymph into PG cells for pheromone biosynthesis in *B*. *mori* [22] and *Eilema japonica* [47], and ACBPs have been functionally confirmed to serve as carriers or cellular deposits for the acyl-CoAs used in pheromone biosynthesis [22]. Therefore, the PG-biased expression of *SlitFATP3* and *SlitACBP2* may indicate that they play a similar role in the pheromone biosynthesis of *S*. *litura*.

Some previous studies identified the presence of chemosensilla on the ovipositor [48, 49], which may function in the chemoreception of plant odors, ovipositor-deterring pheromones, and sex pheromones, suggesting that female moths may receive and transport pheromone compounds or their precursors via their ovipositor [35, 50–52]. This finding further suggests that there may be a feedback loop in the moth’s PG (including the ovipositor) to achieve accurate control of the biosynthesis pathway and release of sex pheromones. To date, many studies have demonstrated the role of OBPs and CSPs in the binding and transportation of hydrophobic molecules, including plant volatiles, sex pheromones and their precursors [50, 53–57]; therefore, these two proteins appear to be essential for the sex pheromone biosynthesis pathway. Similarly to other moths [32, 34, 35], we identified a total of 39 genes in the *S*. *litura* PG, including 25 *OBP* and 14 *CSP* genes, but only 4 genes (*SlitOBP6*, *SlitOBP11*, *SlitCSP3*, and *SlitCSP14*) showed PG-biased expression, indicating that these genes may play important roles in the binding and transport of sex pheromone compounds and plant volatiles.

In conclusion, through sequencing and transcriptome analyses, we obtained an extensive set of putative genes that may be related to the biosynthesis and transport of the sex pheromone of *S*. *litura*. As the first step towards understanding the functions of these genes, we conducted a comprehensive and comparative examination of gene expression patterns and conducted a phylogenetic analysis with sequences of other species. We identified a number of genes with PG-biased or specific expression, indicating their involvement in the biosynthesis and transport of the sex pheromone. Further studies are needed to explore the functions of these genes with integrated functional studies.

## Materials and Methods

### Insects Rearing

*S*. *litura* were reared on an artificial diet comprising wheat germ flour and soybean flour, and were sexed as pupae and kept separately in cages for eclosion. The rearing conditions were 27°C, with a 14-h light:10-h dark photoperiod, and 65 ± 5% relative humidity. Adults were provided with a cotton swab dipped in 10% honey solution that was renewed daily.

### Tissue Collection

For transcriptome sequencing, 20–25 PGs (with the ovipositor) were collected from 3-day-old virgin female adults at 6–7 h into the scotophase, since 3-day-old moths show particularly high mating activity [25]. For the tissue expression study, 25–30 female antennae (FA), 25–30 male antennae(MA), 20–25 PGs (with the ovipositor), 10–15 whole insect body without PGs(B), and 10–15 whole insect body without PGs and antennae (Bo) were also collected under the same conditions. All samples were immediately frozen and stored at −70°C until use.

### cDNA Library Construction

Total RNA was extracted using TRIzol reagent (Invitrogen, Carlsbad, CA, USA). cDNA library construction and Illumina sequencing of the samples were performed at Novogene Bioinformatics Technology Co., Ltd. (Beijing, China). The mRNA was purified from 3 μg of total RNA using oligo (dT) magnetic beads and fragmented into short sequences in the presence of divalent cations at 94°C for 5 min. Then, the first-strand cDNA was generated using random hexamer-primed reverse transcription, followed by synthesis of the second-strand cDNA using RNaseH and DNA polymerase I. After end repair and ligation of adaptors, the products were amplified by PCR and purified using the QIAquick PCR Purification Kit to create a cDNA library, and the library quality was assessed on the Agilent Bioanalyzer 2100 system.

### Clustering and Sequencing

The clustering of the index-coded samples was performed on a cBot Cluster Generation System using TruSeq PE Cluster Kit v3-cBot-HS (Illumina) according to the manufacturer’s instructions. After cluster generation, the library preparations were sequenced on an Illumina Hiseq^(TM)^ 2500 platform, and paired-end reads were generated.

### *De novo* Assembly of Short Reads and Gene Annotation

Clean short reads were obtained by removing reads containing adapter, reads containing ploy-N and low quality reads from the raw reads. Transcriptome *de novo* assembly was carried out with these short reads in the assembling program Trinity (r20140413p1) [58, 59] with min_kmer_cov set to 2 by default, and all other parameters at the default settings. The resulting sequences of Trinity were deemed to be unigenes. Unigenes larger than 150 bp were first aligned to protein databases by BlASTX, including Nr, Swiss-Prot, KEGG, and COG (e-value < 10^−5^), to retrieve proteins with the highest sequence similarity to the obtained unigenes along with their protein functional annotations. Then, we used the Blast2GO program [60] to get the GO annotation of the unigenes, and the GO functional classification was obtained using WEGO software [61].

### Expression Abundance Analysis of the Unigenes

The expression abundance of these unigenes were calculated based on the reads per kilobase per million mapped reads (RPKM) method [62], using the formula: RPKM (A) = (10,00,000 × C × 1,000)/(N × L), where RPKM (A) is the abundance of gene A, C is the number of reads that uniquely aligned to gene A, N is the total number of reads that uniquely aligned to all genes, and L is the number of bases in gene A. The RPKM method is able to eliminate the influence of different gene lengths and sequencing discrepancy in the calculation of expression abundance.

### RNA Isolation and cDNA Synthesis

Total RNA was extracted with the SV 96 Total RNA Isolation System (Promega, Madison, WI, USA) following the manufacturer’s instructions, in which DNaseI digestion was included to avoid genomic DNA contamination. RNA quality was checked with a spectrophotometer (NanoDropTM 1000, Thermo Fisher Scientific, USA). The single-stranded cDNA templates were synthesized from 1 μg of total RNA from various tissue samples using the PrimeScript RT Master Mix (TaKaRa, Dalian, China).

### Sequence Analyses

The open reading frames (ORFs) of the putative chemosensory genes were predicted by using ORF Finder (http://www.ncbi.nlm.nih.gov/gorf/gorf.html). Similarity searches were performed using the NCBI-BLAST network server (http://blast.ncbi.nlm.nih.gov/). Putative N-terminal signal peptides of SlitOBPs and SlitCSPs were predicted by Signal IP 4.1 (http://www.cbs.dtu.dk/services/SignalP/) [63].

### Phylogenetic Analyses

The phylogenetic trees were constructed for phylogenetic analyses of SlitOBPs, SlitCSPs, SlitDes and SlitFAR, based on these genes (the signal peptides of sequences had been removed of the putative chemosensory genes) and the sequences of other insects. The OBP dataset contained 25 sequences from *S*. *litura*, 19 from *Manduca sexta* [37, 64], 15 from *S*. *littoralis* [64], 23 from *Sesamia inferens* [65], and 43 from *B*. *mori* [66]. The CSP dataset contained 14 sequences from *S*. *litura*, 14 from *M*. *sexta* [37], 9 from *S*. *littoralis* [64], 13 from *S*. *inferens* [65], and 14 from *B*. *mori* [67]. The Des dataset contained 12 sequences from *S*. *litura* and 59 from other insects [33]. The FAR dataset contained 13 sequences from *S*. *litura* and 56 from other insects [33]. The names and accession numbers of the genes used for phylogenetic tree construction are listed in S2 Table. Amino acid sequences were aligned with ClustalX 1.83 [68], and unrooted trees were constructed by MEGA5.0 [69] using the neighbor-joining method, with Poisson correction of distances. Node support was assessed using a bootstrap procedure base on 1000 replicates.

### Reverse Transcription-PCR Analyses

Gene-specific primers across the ORFs of predicted chemosensory genes were designed using Primer Premier 5.0 (PREMIER Biosoft International, CA, USA). The sequences of these primers are listed in S3 Table. PCR experiments including negative controls (no cDNA template) were carried out under the following conditions: 94°C for 4 min, 30–35 cycles at 94°C for 30 sec, 60°C for 30 sec, and 72°C for 40 sec, and final extension for 10 min at 72°C. The reactions were performed in a total volume of 25 μL, containing 12.5 μL of 2×EasyTaq PCR SuperMix (TransGene, Beijing, China), 0.4 μM for each primer, 1 μL of sample cDNA (15 ng/μL), 9.5 μL of sterilized H_2_O. PCR products were analyzed by electrophoresis on 1.5% w/v agarose gel in TAE buffer (40 mM Tris-acetate, 2 mM Na_2_EDTA·H_2_O). The gene encoding *S*. *litura* glyceraldehyde-3-phosphate dehydrogenase (*SlitGAPDH*) (GenBank Accession No.: HQ012003.2) was used as a reference gene for checking the integrity of the cDNA template. Each PCR reaction was done at least twice.

### Quantitative Real Time-PCR Validation

The expression profile of 16 putative genes with PG-enriched or specific expression was evaluated to validate the accuracy of the RT-PCR results using quantitative real time-PCR (qPCR) experiments. The qPCR was performed on an ABI 7300 (Applied Biosystems, Foster City, CA, USA) using a mixture of 10 μL 2× SYBR Green PCR Master Mix, 0.4 μL each primer (10 μM), 2.5 ng of sample cDNA, and 6.8 μL sterilized ultrapure H_2_O. The reaction programs were at 95°C for 30 sec, followed by 40 cycles of 95°C for 5 sec, and 60°C for 31 sec. The results were analyzed using the ABI 7300 analysis software SDS 1.4. The qPCR primers (S3 Table) were designed using Beacon Designer 7.9 (PREMIER Biosoft International, CA, USA). The mRNA levels were measured by qPCR using SYBR Premix ExTaq (TaKaRa, Dalian, Liaoning, China). Subsequently, fluorescence was measured throughout a 55–95°C melting curve in order to detect a single gene-specific peak and to check the absence of primer dimer peaks; single and discrete peaks were detected for all primers tested. Negative controls were non-template reactions (replacing cDNA with H_2_O).

The expression levels of 16 genes were calculated relative to the reference gene *SlitGAPDH* (GenBank Accession No.: HQ012003.2) and *SlitEF* (elongation factor–1 alpha) (GenBank Accession No.: DQ192234.1) using the Q-Gene method in the Microsoft Excel-based software Visual Basic [70, 71] For each sample, three biological replications were performed with each biological replication measured in three technique replications.

### Statistical Analyses

Data (mean ± SE) form various samples were subjected to one-way nested analysis of variance (ANOVA) followed by the least significant difference test (LSD) for mean comparison, and two-sample analysis was performed by the Student *t*-test using SPSS Statistics 17.0 software (SPSS Inc., Chicago, IL, USA).

## Supporting Information

S1 Fig Phylogenetic tree of insect odorant binding protein (OBP). The *S*. *litura* translated genes are shown in blue. Accession numbers are given in S2 Table. The tree was constructed with MEGA5.0, using the neighbour-joining method. Values at the nodes are results of bootstrap with 1000 replicates.
(TIF)

S2 Fig Phylogenetic tree of insect chemosensory protein (CSP). The *S*. *litura* translated genes are shown in blue. Accession numbers are given in S2 Table. The tree was constructed with MEGA5.0, using the neighbour-joining method. Values at the nodes are results of bootstrap with 1000 replicates.
(TIF)

S1 Table The Blastx match of *S*. *litura* top20 genes. (XLS)

S2 Table Accession numbers for amino acid sequences of Dess, FARs, OBPs and CSPs used in phylogenetic analyses. (XLS)

S3 Table Primers used for RT-PCR and qPCR. (XLS)
