# Supplementary figures and images for: Identification and Expression Profiles of Sex Pheromone Biosynthesis and Transport Related Genes in Spodoptera litura
Source: PLoS One. 2015 Oct 7;10(10):e0140019. doi: 10.1371/journal.pone.0140019 (PMC4596838; doi:10.1371/journal.pone.0140019)

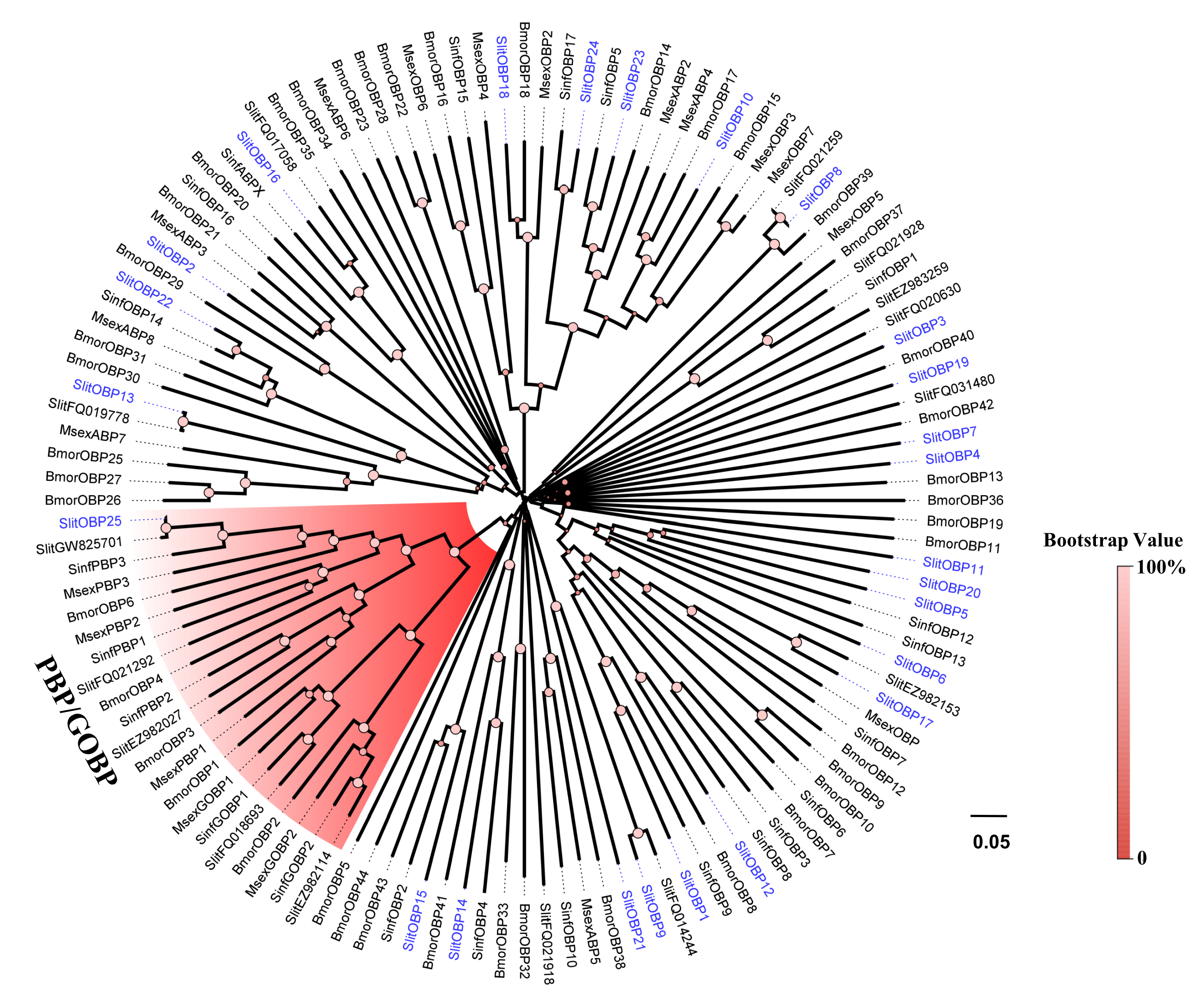

Supplement: S1 Fig — The S. litura translated genes are shown in blue. Accession numbers are given in S2 Table. The tree was constructed with MEGA5.0, using the neighbour-joining method. Values at the nodes are results of bootstrap with 1000 replicates. (TIF) [file pone.0140019.s001.tif]

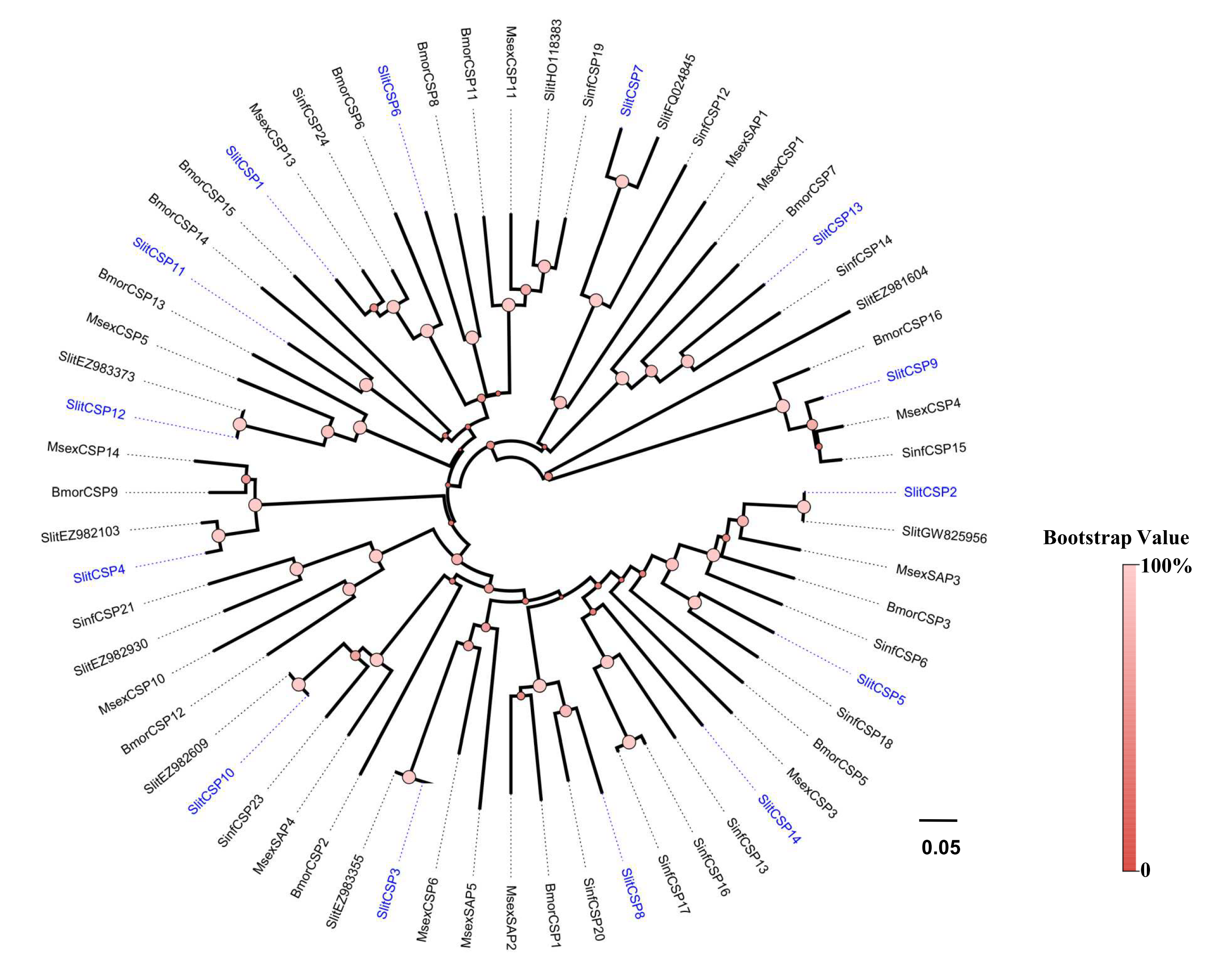

Supplement: S2 Fig — The S. litura translated genes are shown in blue. Accession numbers are given in S2 Table. The tree was constructed with MEGA5.0, using the neighbour-joining method. Values at the nodes are results of bootstrap with 1000 replicates. (TIF) [file pone.0140019.s002.tif]
